# Comprehensive epidemiological evaluation of ruminant brucellosis and associated risk factors in some Egyptian Governorates

**DOI:** 10.14202/vetworld.2024.2780-2796

**Published:** 2024-12-14

**Authors:** Nesreen Allam Tantawy Allam, Mahinour Ezzeldin Abdelsalam, Hend I. Elsharkawy, Mai Mohamed Kandil, Amany Mohamed Mohamed Mohamed, Fatma Ali, Mohamed A. Gebely, Safaa Y. Nour, Doaa Sedky, Mona Ebrahim Hussien Abd El-Gawad, Hoda M. Zaki, Nazek Al-Gallas, Amal M. Aboelmaaty, Mona Mohamed Sobhy, Nagwa Sayed Ata, Marwa Salah Abdel-Hamid, Ghada A. Badawy

**Affiliations:** 1 Department of Parasitology and Animal Diseases, Veterinary Research Institute, National Research Centre, 33 El Buhouth Street, Dokki, P.O. Box: 12622, Cairo, Egypt; 2 Department of General Biology, Center of Basic Sciences, Misr University for Science and Technology, Al Motamayez District, 6^th^ of October, Giza, Egypt; 3 Brucella Reference Laboratory, Animal Health Research Institute, Agricultural Research Center, P.O. Box 264-Giza, Cairo, 12618, Egypt; 4 Department of Microbiology and Immunology, Veterinary Research Institute, National Research Centre, 33 El Buhouth Street, Dokki, P.O. Box: 12622, Cairo, Egypt; 5 Department of Physiology, Faculty of Veterinary Medicine, Aswan University, Egypt; 6 Animal Medicine Department, Faculty of Veterinary Medicine, Aswan University, Egypt; 7 Cytogenetics and Animal Cell Culture Lab., National Gene Bank, Agriculture Research Center, 9 Gamaa Street, Giza, Cairo, Egypt; 8 Department of Biology, Faculty of Science, University of Hafr Al-Batin, P.O. Box: 1803, Hafr Al-Batin, 31991, Kingdom of Saudi Arabia; 9 Water and Food Control Lab., National Center of Salmonella, Shigella, Vibrio, E. coli-Enteropathogens, Institute Pasteur de Tunis, Tunis; 10 Department of Reproduction, Veterinary Research Institute, National Research Centre, 33 El Buhouth Street, Dokki, P.O. Box: 12622, Cairo, Egypt; 11 Department of Reproductive Diseases, Animal Reproduction Research Institute, Agricultural Research Center, Al-Haram, Giza, Egypt; 12 Department of Microbial Biotechnology, Genetic Engineering and Biotechnology Research Institute, University of Sadat City; 13 Department of Botany, Faculty of Science, El-Fayoum University, Fayoum, 63514, Egypt; 14 Department of Biology, Faculty of Science, University of Tabuk, Umluj 46429, Kingdom of Saudi Arabia

**Keywords:** abortion, acute phase proteins, AMOS-PCR, body condition score, *Brucella melitensis*, Egypt, genotoxicity, mastitis, oxidative stress, prevalence, trace elements

## Abstract

**Background and Aim:**

Brucellosis contributes to significant economic losses due to abortion, weak newborns, infertility, and up to 20% reductions in milk yield in carrier animals. This study aimed to estimate the prevalence of ruminant brucellosis in six Egyptian governorates. This study aimed to estimate the prevalence of ruminant brucellosis and evaluate the risk factors regarding the epidemiological status, highlighting the importance of early carrier detection for the success of control programs.

**Materials and Methods:**

A total of 3000 ruminants were investigated. Blood and serum samples were collected for routine hemato-biochemical analysis (complete blood picture and metabolic panel). In addition, genotoxicity analysis was performed, whereas tissue samples were collected for histopathological analysis. The buffered acidified plate antigen test (BAPAT), Rose Bengal plate test (RBPT), and complement fixation test (CFT) were used for serological diagnosis of brucellosis. The obtained bacterial colonies were typed using *Brucella abortus*-*, melitensis*-, *ovis*-, and *sui*s-polymerase chain reaction (AMOS-PCR), depending on the variability of the *IS711* fragment among *Brucella* spp. Serum trace elements, oxidative stress, and acute phase proteins were compared according to body condition score (BCS) and clinical condition images within the study population.

**Results:**

Mastitis and abortion were the key recorded symptoms (9.966%, 299/3000 and 6%, 180/3000, respectively); however, symptomless individuals were predominant (82.9%, 2487/3000). Blood lymphocytosis was prominent even in asymptomatic animals. Nutritional and food conversion conditions were defined as low, moderate, or high BCS. *Brucella* overall seropositivity by BAPAT, RBPT, and CFT was 6.1% (182/3000), 5.6% (168/3000), and 5.1% (154/3000) in ruminant species within the included governorates, respectively. Upon diagnosis, 154 seropositive cases developed 93 bacterial isolates and a 731-bp PCR fragment whose sequences confirmed *Brucella melitensis* biovar 3. Serum metabolic and biochemical profiles, acute phase proteins, trace elements, and oxidative stress concentrations were indicative of loss of functionality in the liver and kidneys, malnutrition and malabsorption syndrome, and DNA damage, particularly in the low-BCS groups (p < 0.0001). Granulomatous lesions were most prominent in the lymph nodes, spleen, uterus, and udder of the dams, while placental multifocal necrosis with thrombosis was recorded in aborted fetuses. There were 8 types of chromosomal aberrations detected in peripheral white blood cells. The highest frequency was for dicentric aberrations 0.025% (25/1000), whereas the lowest 0.009% (9/1000) was for acentric, ring, fusion, and polyploidy. The difference between species was significant for BCS; 14.2% in low-BCS cattle and camels and 8.4% in high-BCS buffaloes.

**Conclusion:**

*B. melitensis* biovar 3 is prevalent in Egypt. Mixed-rearing systems are the main risk factors for interspecies transmission among ruminants. The difficulty in accurately diagnosing all infected animals, particularly carriers, is a major limitation of eradication and control programs. Different biomarkers could be indicators and/or sensors for performance and/or infectivity conditions in animal herds; however, they require further optimization. Early detection using molecular technologies, highly descriptive, quantitative, sensitive, and specific methods, as alternatives to serological diagnosis (CFT, BAPAT, and RBT), is urgently needed to enhance the efficiency of brucellosis-specific prophylaxis. Such a comprehensive procedure is the World Organization for Animal Health dependent decision.

## Introduction

Despite 170 years since its first description in Malta, brucellosis remains a notorious zoonosis with adverse public health and animal disease consequences. Hence, the continuous dissemination of pathogens obstructs the trade of animals and their bioproducts [1–3]. *Brucella melitensis*, *Brucella suis*, *Brucella abortus*, and *Brucella canis* are the most virulent species in both humans and animals, leading to persistent infection of the host reticuloendothelial system. The other identified member species were *Brucella ovis*, *Brucella neotomae*, *Brucella ceti*, *Brucella pinnipedialis*, *Brucella microti*, *Brucella inopinata*, *Brucella papionis*, and *Brucella vulpis*. Hence, a wide range of non-reproductive and non-specific symptoms, including endophthalmitis, lymphadenitis, discospondylitis, chronic uveitis, fatigue, decreased appetite, and weight loss, are common conditions that necessitate differential diagnostic efficiency [4–7]. Notably, this may also remain unapparent in animal herds. Most cases are caused by severe or fatal complications associated with other infections due to immune failure [8]. Persistence is preserved by evading the host immune system during intracellular parasitism, which is preferred in trophoblasts, fetal tissues, and male and female reproductive organs, thereby distressing the economic effects on livestock production [4, 6, 9, 10]. Regarding large-scale investigations, the laboratory diagnosis of brucellosis is mainly based on serological tests [2, 11]. However, the similarity of the O-antigenic side chain of *Brucella* lipopolysaccharide (LPS) to that of other microbes, particularly *Yersinia enterocolitica* O:9, restricts the specificity of serological diagnostic procedures [12]. Moreover, the culture of *Brucella* is time-consuming, expensive, has low sensitivity, poses difficulty in the interpretation of results, and requires biohazard conditions (LB3) for handling highly contagious materials [13]. Owing to the disadvantages of serological and bacteriological methods, new technologies based on molecular biology during polymerase chain reaction (PCR) have been introduced into routine diagnostics [6]. The earliest PCR assays used for *Brucella* identification concentrated on genus-specific loci, which have been proven to be sufficient for diagnosing brucellosis in humans and in food product contamination [14]. However, brucellosis eradication programs are typically species-specific, and the associated regulatory actions are also species-dependent [2]. Moreover, differential assays are useful for epidemiological investigations and identifying the source of infection [4, 6, 9, 10, 15]. *Abortus*, *melitensis*, *ovis*, and *suis* polymerase chain reaction (AMOS-PCR) was the first molecular assay to detect *Brucella* chromosome polymorphism arising from species-specific localization of the *IS711* insertion sequence. AMOS-PCR could differentiate between *B. abortus*, *B. melitensis*, *B. ovis*, and *B. suis* in the investigated samples [16]. In Egypt, brucellosis was first reported between 1939 and 1942. *B. melitensis* biovar 3 is the most widely circulating *Brucella* species in ruminants, followed by *B. abortus* biovar 1 and *B. suis* biovar 1 and 2 [17–19]. Recently, DNA from *B. canis* was detected in the buffy white coats of stray and owned dogs in Egypt [4–6, 9, 10, 15]. Unfortunately, most *Brucella* research on cloven-hoofed species has focused on bovines to determine the status of *B. melitensis*, *B. abortus*, and *B. suis* [5, 6]. However, individual and/or large-scale mixed farming of ruminant spp. increases cross-species affinity [2]. There has been no attempt to determine brucellosis status in other species, namely sheep, goats, camel, and canine predominantly through cross-species transmission of brucellosis [6]. Therefore, the proposed outcomes could be achieved through a sound epidemiological assessment of risk factors related to the spread and persistence of brucellosis in endemic and non-endemic areas and free areas [2, 4].

Animal management and environmental factors are the main risk factors for brucellosis [2]. Animal factors include age, sex, parity, breed, retained placenta, abortion, and milking system [20]. Intraspecies transmission of *Brucella* spp. is facilitated by ingestion, inhalation, mating, and direct contact with infectious biohazards [4]. Management-related risks include the production system, breeding and disinfection practices, herd size and density, vaccination, new arrival screening for *Brucella*, and disease awareness outcomes [21, 22]. Environmental risk factors are climate, ecology, humidity, temperature, sunlight duration, rotation of grazing pasture, disinfection practices, and proximity to wildlife [22, 23]. Inadequate policies, limited resources, lack of awareness of the disease, and/or ineffective control programs have been the reasons for inefficient disease eradication in developing countries [3, 13, 24].

This study aimed to estimate the prevalence of ruminant brucellosis in El-Behera, El-Sharkia, Kafr El-Sheikh, El-Monefia, El-Giza, and Aswan governorates of Egypt. The epidemiological update determined *Brucella* species circulating among ruminants in the studied species on a bacteriological and molecular basis using the multiplex PCR (mPCR) method. In addition, we investigated the histopathological changes in the internal organs and placenta associated with abortion in dams infected with *Brucella* and evaluated the risk factors associated with this disease.

## Materials and Methods

### Ethical approval

All clinical samples were collected according to a standard procedure without harming the animals and condemnation of biohazardous materials was according to World Organization for Animal Health (WOAH) guidelines [2]. The experimental procedures were conducted in accordance with the guidelines and were approved by the Ethical Committee for Medical Veterinary Research at the Genetic Engineering and Biotechnology Research Institute, Sadat City University, as well as the Animal Care Guidelines of the General Organization for Veterinary Services (GOVS), Egypt during all test and slaughter protocol, quarantine, brucellosis retesting, and condemnation of infected carcasses (approval no.: IACUC-GEBRI-USC-122019).

### Study period and location

This study was conducted from June 2021 to August 2023 on 3000 ruminants of different species infected with *Brucella* in farms under quarantine by the GOVS. The samples were collected from six governorates of Egypt: El-Behera (30.61°N 30.43°E), Kafr El-Shiekh (31.3°N 30.93°E), El-Sharkia (30.7°N 31.63°E), El-Monufia (30.52°N 30.99°E), El-Giza (29.26°N 29.67°E), and Aswan (23.59°N 32.82°E).

### Selection of animals

A two-stage random sampling method was used. The first stage involved the random selection of farms, and the second stage involved the random selection of animals from those farms that met our inclusion criteria: adults with clinical symptoms and in contacts without clinical symptoms kept under quarantine by GOVS during *Brucella* test and slaughter procedures. Therefore, 100 individuals/species were included, resulting in 3000 sampled individuals. The animal species included cattle (600), buffaloes (600), sheep (600), goats (600), and camels (600). Selected animals were classified according to their body condition score (BCS), which ranged from 2.5 to 3 [25]. In addition, the recorded clinical symptoms included mastitis, abortion, and asymptomatic status. The management system for the investigated animal population consisted of an open housing system. Animals of different ages were overcrowded in the open housing system. Moreover, different species with varied conditions (aborted and pregnant), males, and females were housed together under natural environmental conditions and fed *ad libitum* commercial concentrate ration (16% crude protein) and clover (*Trifolium alexandrinum*), with a routine physical examination to check their health and reproductive status [26, 27].

### Samples collection

#### Blood

Three types of blood samples were collected from all animals. Whole blood samples were collected through jugular venipuncture using ethylenediaminetetraacetic acid (EDTA) and heparin for hematological and genotoxicity studies, respectively. All samples were labeled and stored at 4°C until further analysis. The other samples were collected in plain tubes and allowed to clot. Then, the serum was separated and preserved at −20°C until used for serological diagnosis. Strict aseptic precautions were taken by veterinary inspectors during sample collection, and disposable gloves were used to collect each sample.

#### Tissues

Samples were collected from the spleen, lymph nodes, uterus, and udder of seropositive animals and the placenta of aborted animals, considering the total 100 individuals/species. The samples were collected and processed for bacterial isolation, as described by Alton *et al*. [28]. Parts of these specimens were fixed in 10% neutral-buffered formalin for 48 h and embedded in paraffin for histopathological examination.

### Diagnostic methods

#### Serological examination

Sera were diagnosed as *Brucella*-positive using a buffered acidified plate antigen test (BAPAT), Rose Bengal plate test (RBPT), and complement fixation test (CFT), with antigens in the *Brucella* Reference Laboratory, Animal Health Veterinary Research Institute, Egypt. BAPAT was performed using killed *B. abortus* strain 99 antigens at a concentration of 11% in lactate buffer (pH 3.70 ± 0.03). RBPT consisted of killed *B. abortus* strain 99 antigens at a concentration of 8% cells in lactate buffer (pH 3.65 ± 0.05). In contrast, the CFT warm-microtechnique antigen was specific to *B. abortus* biovar 1 strain 1119-3 cells in phenol saline (pH 6.8) at a concentration of 4.5%. All procedures were performed according to Alton *et al*. [28].

#### Bacteriological examination

Tissue samples of seropositive animals slaughtered under the supervision of GOVS were plated on tryptose agar medium with a *Brucella* antibiotic-selective supplement (Oxoid), according to Dadar and Alamain [29]. The plates were incubated at 37°C in a 10% CO_2_ incubator with daily examination for 10 days for growth. Bacteriological standard identification and characterization were phenotypically identified and characterized in the obtained *Brucella* isolates. The identification scheme included colonial morphology, a series of conventional biochemical tests, the addition of 10% CO_2_, thionine, and fuchsin inhibitory dyes required for colony growth, and agglutination with polyclonal monospecific antisera (A, M, and R), as well as Tbilisi (Tb) and Izatnagar (Iz1) for phage typing, according to the recommended procedures by Alton *et al*. [28]. Standard *Brucella* strains were used as positive controls during all identification processes. All previous steps were repeated twice for each sample.

#### Molecular characterization of the isolates

DNA was purified from *Brucella* colonies (Qiagen, Hilden, Germany). The eluted DNA was stored at −20°C until use in mPCR assays depending on the differential diagnostic efficacy of the *IS711* fragment among *Brucella* spp. EmeraldAmp®GT PCR master mix (Takara RR310A kit, Japan) was used with some modifications in the cycling protocol that were primers dependent. After 5 min of heating at 94°C for primary denaturation, each thermal cycle (which was repeated 35 times) involved the following steps: 30 s at 94°C, 1 min at 55°C, and 1 min at 72°C for denaturation, annealing, and extension, respectively. The final extension step was performed for 10 min at 72°C [14]. Primers (Metabion, Germany) used during mPCR are listed in Table-1. PCR products were separated by electrophoresis on 1% agarose gels (Applichem, Germany) in 1× Tris Borate EDTA buffer at 25°C [14]. The Gene-Ruler 100-bp DNA ladder (Fermentas, Germany) was used to determine fragment size. The gel was photographed using a documentation system (ChemiDoc system®Bio-Rad, USA), and the data were analyzed using computer software (Image Lab. Version 4.1®, Bio-Rad).

**Table-1:** Oligonucleotide primer sequences.[14]

Gene	Target agent	Sequence	Amplified product (bp)
*IS711*	*Brucella* spp.	TGCCGATCACTTAAGGGCCTTCAT	*1S711*-specific Primer
	*Brucella abortus*	GACGAACGGAATTTTTCCAATCCC	498
	*Brucella melitensis*	AAATCGCGTCCTTGCTGGTCTGA	731
	*Brucella ovis*	CGGGTTCTGGCACCATCGTC	976
	*Brucella suis*	GCGCGGTTTTCTGAAGGTTCAGG	285

### Hematological profiles

Blood EDTA samples from the investigated animal groups were used in hematological profile analyses. Comparisons were made between the BCS and clinical conditions in all included individuals.

#### Metabolic and biochemical profiles

The serum concentrations of total proteins (Biuret method), albumin, globulin, albumin/globulin (A/G) ratio (Bio-diagnostic Kits, Egypt), and cholesterol were determined (Linear Chemicals Kits, Egypt). The liver enzymes alanine aminotransferase (ALT) and aspartate aminotransferase (AST) were determined using an ultraviolet enzymatic colorimetric method (Linear Chemicals Kits) according to a method described by Allam *et al*. [30]. Serum urea and urea nitrogen levels were also measured (Linear Chemicals Kits). Superoxide dismutase (SOD) and glutathione peroxidase (GPx) levels were estimated as previously reported by Allam *et al*. [30] using commercially available kits (Bio-diagnostic Kits).

#### Trace element and oxidative stress biomarker levels

Nitric oxide (NO), copper, iron, and zinc levels were estimated as previously reported by Abdelnaby *et al*. [31] using commercially available kits (Bio-diagnostics) to compare the profiles between the *Brucella* seropositive and seronegative animal groups. The sensitivity of the assay was 0.225 mmol/L. The intra- and inter-assay variation coefficients were 5.3% and 6.9%, respectively.

#### Acute phase proteins and inflammatory biomarkers

Quantitation of both haptoglobin (HP3222) and fibrinogen was performed using immunoturbidimetric assays according to the manufacturer’s instructions (Benbiochemical Enterprise and Salucea, The Netherlands), and as described by Matson *et al*. [32] and Ernst and Resch [33]. The sensitivity limits for haptoglobin and fibrinogen were 2.9 and 4.5 mg/dL, respectively.

### Histopathological studies

Tissue samples from *Brucella-*positive animals were used to isolate *Brucella* colonies, which were fixed in 10% neutral-buffered formalin for 2 days. The samples were then processed using the paraffin embedding method (dehydrated, cleared, embedded in paraffin), sectioned at 5–7 μm, and then stained with hematoxylin and eosin according to Suvarna *et al*. [34]. Microscopic examination of the stained slides was performed for the comparative pathological evaluation of infection courses in association with the *Brucella*-free samples.

### Genotoxicity assessment

#### Cell culture

Lymphocytes were separated from the samples and cultured using the whole-blood microculture technique described by Abd El-Gawad *et al*. [35] and Kenthao *et al*. [36].

#### Cell harvesting and slide preparation

When the harvest time approached, at 71 h of incubation, 0.2 mL of colcemid was added to the cell culture, mixed gently, and then further incubated for 60 min at 37°C. The blood sample was centrifuged at 112× *g*for 10 min, and the supernatant was discarded. The cells were suspended in 10 mL of hypotonic solution (0.075 M KCl) and incubated for 60 min at 37°C. The mixture was centrifuged at 112× *g* for 10 min, and the supernatant was discarded. The cells were fixed in fresh cold fixative (3 absolute methyl alcohol: 1 glacial acetic acid), which was gradually added up to 8 mL before centrifuging again at 112× *g* for 10 min, and the supernatant was discarded. This fixation step was repeated until the supernatant became clear. The pellet was then mixed with 1 mL of fixative. The cell mixture was dropped onto a clean and cold slide using a micropipette. This was followed by drying the slide on a hot plate [35, 37].

#### Microscopic examination

Chromosomal examinations were performed using a vertical fluorescence microscope (Leica DM2500, Leica Microsystems, Germany) equipped with a cooled monochromatic digital camera (Leica DFC340FX, Leica Microsystems, Germany). Twenty cells with clearly observable and well-spread chromosomes were examined and photographed using oil immersion at 100× magnification. Fifty well-spread metaphase cells were scored per slide and screened for chromosomal abnormalities; significance was estimated using Student’s *t*-test (p < 0.05), according to Allam *et al*. [30].

### Statistical analysis

The results of the diagnostic tests classified the studied population into infected and non-infected males and females. A simple one-way analysis of variance (ANOVA) was then performed to study the effect of class on all the studied parameters. Samples positive for *Brucella* were primarily classified according to low-, moderate-, and high-BCS groups. The clinical symptoms recorded in these seropositive reactors were then classified into three groups: those showing mastitis and abortion and those who were symptomless. Data on hematological parameters, metabolic parameters, and immunological markers were analyzed and presented as mean and standard deviation. Statistical analyses were performed using SPSS for Windows (SPSS Inc., USA) [38]. Data were analyzed using a simple one-way ANOVA and Duncan’s multiple range test to differentiate between significant means at p < 0.05 [39].

## Results

### Clinical symptoms and BCS

**Figure-1:**
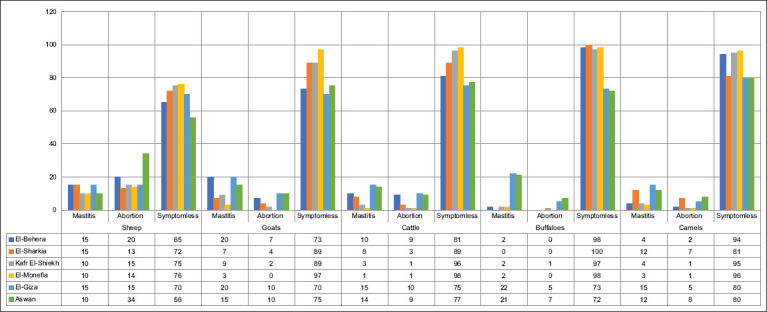
Clinical symptoms recoded in studied animals’ species.

**Figure-2:**
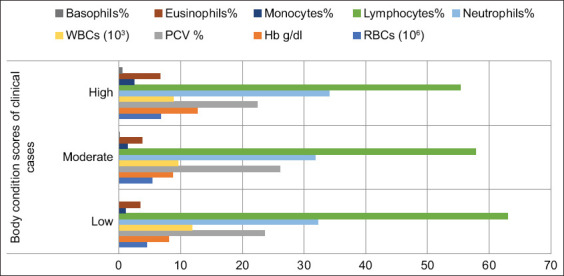
Hematological profiles with regard to body condition scores among the studied *Brucella* infected species.

Mastitis and abortion were the most common symptoms, recorded at 9.966% (299/3000) and 6% (180/3000), respectively (Figure-1). However, symptom-free animals predominated among the investigated groups, accounting for 82.9% (2487/3000). In addition, the nutritional and food conversion conditions within the study groups were expressed as BCS; hence, three levels were recorded: low, moderate, and high. The hematological profiles of the three BCS levels in the studied animal groups are illustrated in Figure-2.

### Serological diagnosis

The results of the seroprevalence of brucellosis in the studied animal species and investigated governorates are presented in Tables-2–4. BAPAT, RBPT, and CFT detected 6.1% (182/3000), 5.6% (168/3000), and 5.1% (154/3000) positive, respectively (Tables-2–4). BAPAT detected 7.3% (44/600), 4.7% (28/600), 9% (54/600), 6.7% (40/600), and 2.7% (16/600) positive cattle, buffaloes, sheep, goat, and camels, respectively (Table-2). RBPT detected 6.5% (39/600), 4.3% (26/600), 8.7% (52/600), 6% (36/600), and 2.5% (15/600) positive cattle, buffaloes, sheep, goat, and camels, respectively (Table-3). CFT detected 5.8% (35/600), 4% (24/600), 8% (48/600), 5.5% (33/600), and 2.3% (14/600) positive cattle, buffaloes, sheep, goat, and camels, respectively (Table-4).

**Table-2:** Seropositivity of brucellosis in examined animals from different governorates in Egypt using Buffered Acidified Plate Antigen Test (BAPAT).

Governorate	Cattle		Buffalo		Sheep		Goat		Camel		Total	
					
	No.	%	No.	%	No.	%	No.	%	No.	%	No.	%
El-Behera	6/100	6	4/100	4	8/100	8	6/100	6	2/100	2	26/500	5.2
El-Sharkia	5/100	5	5/100	5	7/100	7	5/100	5	2/100	2	24/500	4.8
Kafr El-Sheikh	5/100	5	4/100	4	10/100	10	6/100	6	2/100	2	27/500	5.4
El-Monefia	9/100	9	3/100	3	7/100	7	7/100	7	3/100	3	29/500	5.8
El-Giza	9/100	9	6/100	6	10/100	10	7/100	7	3/100	3	35/500	7
Aswan	10/100	10	6/100	6	12/100	12	9/100	9	4/100	4	41/500	8.2
Total	44/600	7.3	28/600	4.7	54/600	9	40/600	6.7	16/600	2.7	182/3000	6.1

**Table-3:** Seropositivity of brucellosis in examined animals from different governorates in Egypt using Rose Bengal Plate Test (RBPT).

Governorate	Cattle		Buffalo		Sheep		Goat		Camel		Total	
					
	No.	%	No.	%	No.	%	No.	%	No.	%	No.	%
El-Behera	6/100	6	4/100	4	7/100	7	4/100	4	2/100	2	23/500	4.6
El-Sharkia	4/100	4	4/100	4	7/100	7	5/100	5	2/100	2	22/500	4.4
Kafr El-Sheikh	5/100	5	4/100	4	10/100	10	5/100	5	2/100	2	26/500	5.2
El-Monefia	8/100	8	3/100	3	7/100	7	7/100	7	3/100	3	28/500	5.6
El-Giza	8/100	8	6/100	6	10/100	10	7/100	7	3/100	3	34/500	6.8
Aswan,	8/100	8	5/100	5	11/100	11	8/100	8	3/100	3	35/500	7.0
Total	39/600	6.5	26/600	4.3	52/600	8.7	36/600	6	15/600	2.5	168/3000	5.6

**Table-4:** Seropositivity of brucellosis in examined animals from different governorates in Egypt using Complement fixation test (CFT).

Governorate	Cattle		Buffalo		Sheep		Goat		Camel		Total	
					
	No.	%	No.	%	No.	%	No.	%	No.	%	No.	%
El-Behera	5/100	5	4/100	4	7/100	7	4/100	4	2/100	25	22/500	4.4
EL- Sharkia	4/100	4	4/100	4	6/100	6	5/100	5	2/100	2	21/500	4.2
Kafrelsheikh	5/100	5	4/100	4	8/100	8	5/100	5	2/100	2	24/500	4.8
El-Monefia	6/100	6	3/100	3	7/100	7	6/100	6	2/100	2	24/500	4.8
El- Giza	7/100	7	5/100	5	10/100	10	6/100	6	3/100	3	31/500	6.2
Aswan,	8/100	8	4/100	4	10/100	10	7/100	7	3/100	3	32/500	6.3
Total	35/600	5.8	24/600	4	48/600	8	33/600	5.5	14/600	2.3	154/3000	5.1

### Bacterial isolates

Serologically positive cases (n = 154/3000) were subjected to bacterial isolation using specific media. A total of 93 *Brucella* isolates were obtained (Table-5), including 19, 15, 29, 20, and 6 bacterial colonies developed from the samples of serologically confirmed positive cases of cattle, buffaloes, sheep, goats, and camels, respectively (Table-5).

**Table-5:** Number of *Brucella* strains isolated from tissue samples from serologically positive animals.

Governorate	Cattle	Buffalo	Sheep	Goat	Camel	Total
El-Behera	3/5	3/4	3/7	1/4	1/2	11/22
El-Sharkia	2/4	1/4	2/6	3/5	0/2	8/21
Kafr El-Sheikh	2/5	3/4	6/8	4/5	1/2	18/24
El-Monefia	4/6	2/3	6/7	4/6	1/2	19/24
El-Giza	3/7	3/5	7/10	3/6	2/3	18/31
Aswan	5/8	3/4	5/10	5/7	1/3	19/32
Total	19/35	15/24	29/48	20/33	6/14	93/154

### Molecular characterization of isolates

**Figure-3:**
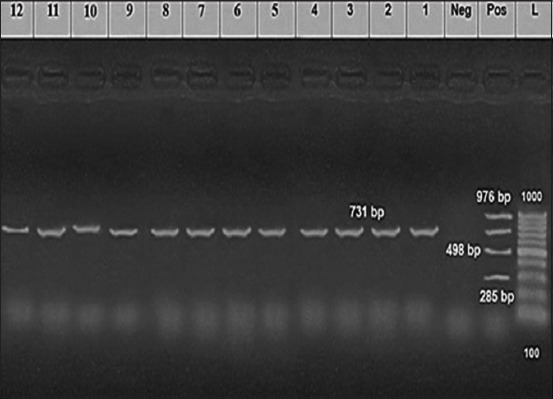
Agarose gel electrophoresis of polymerase chain reaction products amplified from positive samples for *Brucella* spp. Lanes; (L): Marker; Pos: Positive control; Neg: Negative control. Lanes (1–12): showing 731 bp fragments indicative of *Brucella melitensis* infections.

Specified 731-bp molecular weight bands were documented in the lanes of PCR-positive samples compared with DNA marker bands (Figure-3). The specificity and taxonomy of the designated *Brucella* species were determined by fragments’ sequencing using specific primers. The BLASTN of the FASTA files for each sequence in the GenBank database were confirmed to be *B. melitensis* biovar 3 spp. colonies.

### Hematological profiles related to clinical condition and BCSs

**Figure-4:**
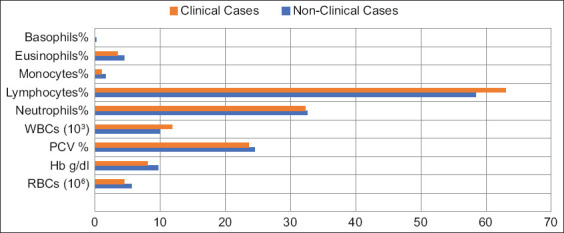
Hematological profiles of the clinical conditions recorded in studied population.

As shown in Figures-2 and 4, a linear increase in the number of red blood cells/million (p = 0.0001) was observed in animals infected with *Brucella* with low-BCS compared with those with high-BCS (4.47 ± 0.34 and 6.81 ± 0.49, respectively). Moreover, hemoglobin concentrations increased (p = 0.0001) in animals with high-BCS compared with those with low- or moderate-BCS (9.64 ± 2.58, 8.06 ± 1.60, and 8.68 ± 1.08, respectively). On the other hand, the packed cell volume of the moderate-BCS group was higher (26.08 ± 1.87, p = 0.011) than those with high-BCS (22.46 ± 7.99), with no differences between animals with low- (23.57 ± 4.29) or high-BCS. White blood cell count was higher (p = 0.059) in the low-BCS group than that of the high-BCS group (11.85 ± 3.66 and 8.85 ± 3.85, respectively), with no differences between the moderate- (9.60 ± 4.85) and high-BCS (11.85 ± 3.66) groups; moreover, it is decreased linearly (p = 0.059) with the increase in BCS (Figure-2). The numbers of monocytes (p = 0.0001), eosinophils (p = 0.012), and basophils (p = 0.0001) increased in the high-BCS group (6.62 ± 6.46, 6.62 ± 6.46, and 0.20 ± 0.38) compared with the low- (3.43 ± 3.09 and 0.00 ± 0.00) and moderate-BCS (3.74 ± 2.53 and 0.10 ± 0.27) groups, respectively (Figure-4).

The hematological profiles of clinical conditions indicated the effect of infection on blood components compared with non-clinical cases. The most prominent indicator was lymphocytosis, even when the patient was symptomless at any BCSs (Figures-2 and 4).

### Metabolic and biochemical profiles

The total protein and albumin concentrations were significantly different between the groups. The low-BCS group had the highest total protein and globulin concentrations (Table-6). In contrast, the albumin concentration was highest in the high-BCS group (Table-6). In contrast, the A/G ratio showed no significant differences in sera among all BCS groups; however, a slight increase was observed in the low-BCS group (Table-6). Hepatic damage due to infection was determined by estimating total protein and enzyme activities, such as AST and ALT. Elevated serum enzyme levels indicate cellular leakage and loss of functional integrity of the cell membrane in the liver, which were most significant in the low-BCS group (Table-6). The same results were reported for serum urea and urea nitrogen concentrations in the low-BCS group (Table-6). Both parameters are indicators of renal dysfunction. The lowest SOD activity (400.00 ± 70.00 U/mL) was observed in the low-BCS group, whereas the highest was noted in the high-BCS group (540 ± 200.0 U/mL), as shown in Table-6. The same was illustrated by the results of GPx; low-BCS and high-BCS groups had levels of 149.00 ± 30.00 and 30.10 ± 26.30 mU/mL, respectively (Table-6).

**Table-6:** Metabolic and biochemical profiles of studied groups classified according to body conditions scores (Mean ± Standard deviation)

Parameter	Low-BCS	Moderate-BCS	High-BCS
Total proteins g/dL	7.90 ± 0.6*	7.50 ± 0.28	7.00 ± 0.40
Albumin g/dL	4.00 ± 0.2	4.30 ± 0.70	4.90 ± 0.45**
Globulin g/dL	4.00 ± 0.4*	3.50 ± 0.30	3.70 ± 0.35
A/G ratio	1.10 ± 0.1	1.09 ± 0.10	1.00 ± 0.90
AST U/mL	150.50 ± 3.0**	147 ± 3.00	116.30 ± 6.80
ALT U/mL	53.00 ± 2.0*	52.50 ± 1.90	47.50 ± 0.75
Urea g/dL	98.00 ± 20.0**	44.50 ± 1.90	37.00 ± 2.00
Urea nitrogen g/dL	59.00 ± 15.3**	49.00 ± 10.50	17.00 ± 1.00
SOD U/mL	400.00 ± 70.00	425.00 ± 130.00	540 ± 200.0**
GPx mU/mL	30.10 ± 26.30	67.80 ± 67.0	149.00 ± 30.00**

Values have

* and

** were significantly different at *p* < 0.05 and 0.01, respectively. BCS=Body condition score.

### Trace elements and oxidative stress biomarker levels

**Figure-5:**
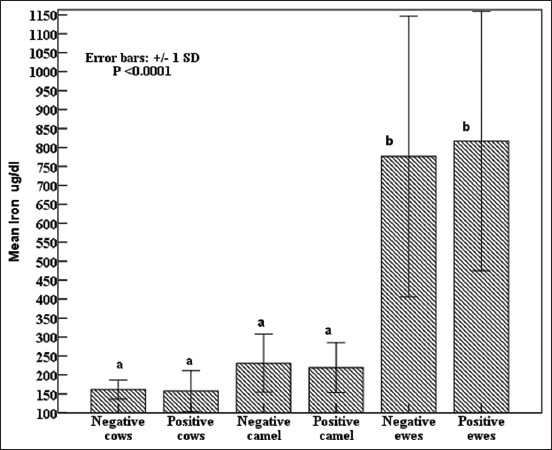
Effects of *Brucella* infection on circulating iron concentrations in cows, camels, and sheep with standard deviation bars at p < 0.0001.

**Figure-6:**
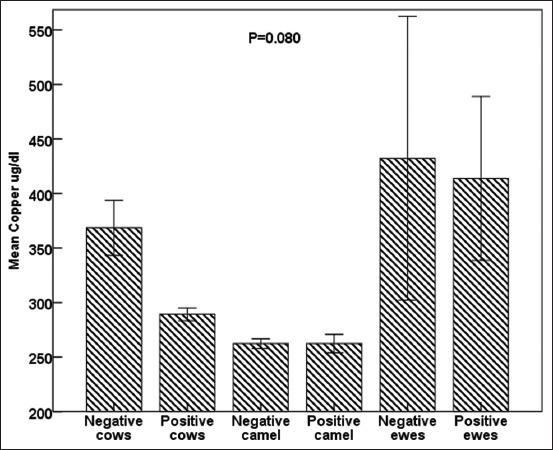
Effects of *Brucella* infection on circulating copper concentrations in cows, camels, and sheep; standard deviation bars, p = 0.080.

**Figure-7:**
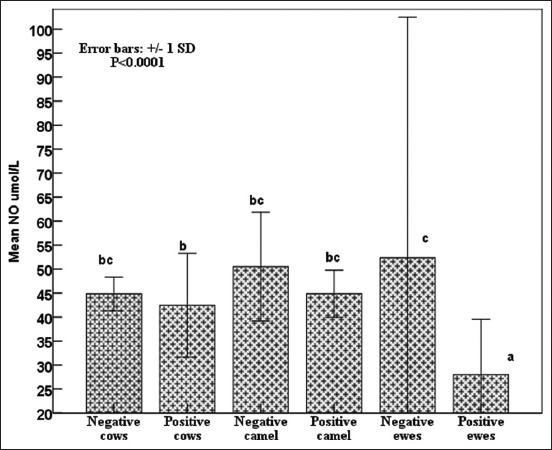
Effects of *Brucella* infection on circulating nitric oxide biomarker concentrations in cows, camels, and sheep; standard deviation bars at p < 0.0001.

Notably, iron concentrations did not vary between seropositive and seronegative groups in all studied species, but both serotypes of sheep had the highest (p < 0.0001) iron concentrations (Figure-5). Copper concentrations tended to be lower in seropositive cows and ewes than in seronegative (Figure-6). All seropositive animals had lower (p < 0.0001) NO concentrations than seronegative animals (Figure-7).

#### Acute phase protein-inflammatory biomarkers

The seropositive group had significantly higher haptoglobin and zinc levels (15.3 ± 17.5 mg/dL and 126 ± 13 μg/dL, respectively) than the seronegative group (9.1 ± 4.7 mg/dL and 104 ± 12 μg/dL, respectively) (p < 0.05). In contrast, the seronegative group had significantly lower fibrinogen concentrations than the seropositive group (168 ± 69 and 303 ± 120 mg/dL, respectively) (p < 0.05).

### Histopathological changes

**Figure-8:**
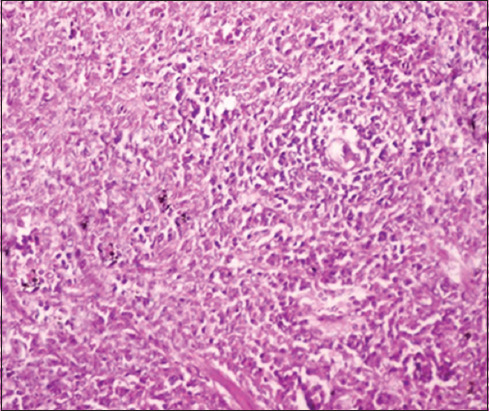
Lymph nodes of a sheep showing a cortex with dilated lymphoid follicles, pale centers, marked depletion of lymphocytes, and proliferation of reticuloendothelial cells, with golden brown patches of irregular shapes of hemosiderosis (H&E, 400×).

**Figure-9:**
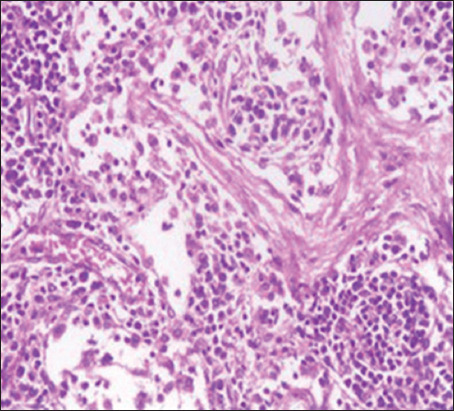
Lymph nodes of a cow showing granulomatous reaction; the predominant cells were macrophages, plasma cells, and lymphocytes (H&E, 400×).

**Figure-10:**
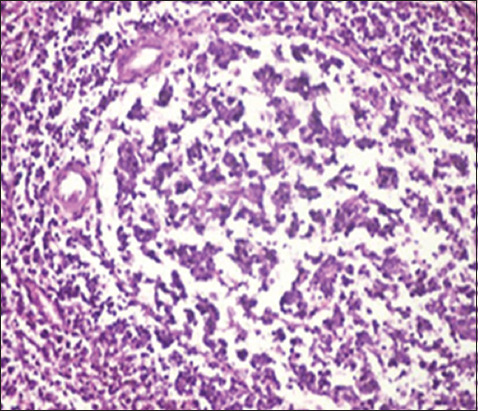
Spleen of a sheep showing degeneration and depletion of lymphocytes in the white pulp. The splenic artery was constricted by protrusion of the endothelial cell lining toward the lumen (H&E, 400×).

**Figure-11:**
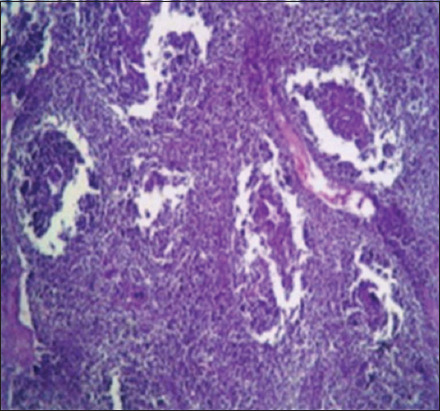
Spleen of a cow showing severe depletion of the lymphoid follicle (H&E, 200×).

**Figure-12:**
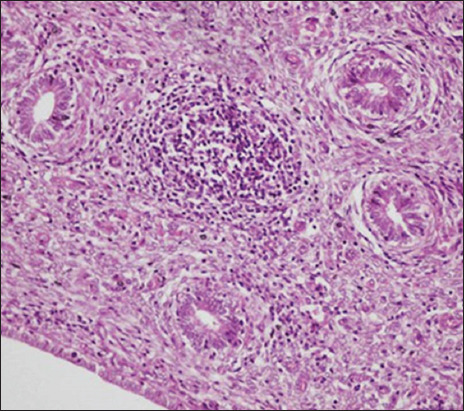
Uterus of a sheep showing endometritis with granulomatous reaction in uterine tissue and chronic endometritis with the proliferation of the fibrous connective tissue stroma, especially around the uterine gland (H&E, 400×).

**Figure-13:**
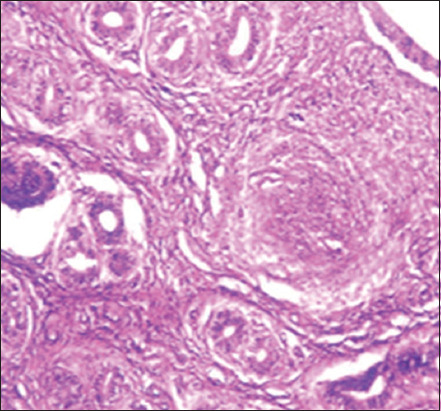
Uterus of a cow showing granulomatous reaction in the uterine parenchyma and some uterine glands showing necrosis and calcification (H&E, 200×).

**Figure-14:**
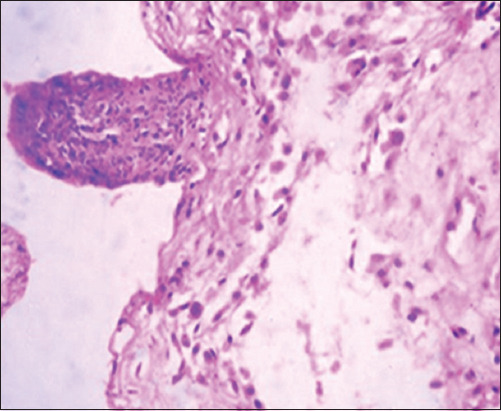
Udder of a cow showing vacuolar degeneration and destruction of epithelial cells lining the glandular acini of the udder with mononuclear cell infiltration (H&E, 200×).

**Figure-15:**
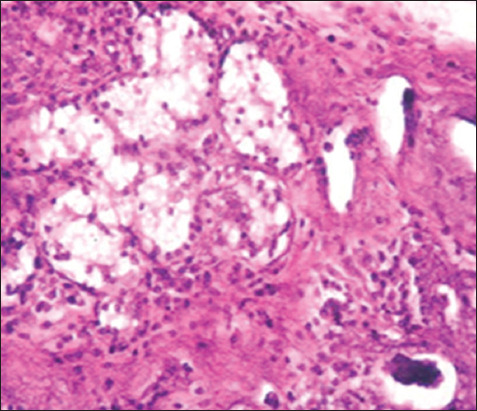
Placenta of an aborted fetus of cow showing necrosis in cotyledonary villi associated with infiltration of mononuclear inflammatory cells (H&E, 200×).

The pathological changes observed in the present study were most prominent in sheep and cattle groups: The lymph node (Figures-8 and 9), spleen (Figures-10 and 11), uterus (Figures-12 and 13), and udder (Figure-14) of the aborted dams and the placenta (Figure-15) of aborted fetuses. Macroscopic or post-mortem (PM) examination of the infected animals revealed enlargement of the spleens with petechial hemorrhage in the splenic capsule in some cases. In other cases, the spleen was firm in texture with whitish necrotic foci. The lymph nodes appeared enlarged and edematous. The uterus was hard to texture, the endometrium appeared congested and edematous, and minute grayish-white foci were observed on the surface of the caruncles.

Histopathological examination of the *Brucella*-infected lymph nodes and spleens in sheep revealed dilated lymphoid follicles with pale centers and marked depletion of lymphocytes (Figure-8). Degeneration and depletion of lymphocytes were observed in the white pulp of the spleen, and the splenic artery was constricted with protrusion of endothelial cells lining toward the lumen (Figure-10). An increase in the number of plasma cells was observed in the medullary sinuses (Figure-9). Microscopic examination of the spleens of infected cows revealed blood vessel wall thickening and perivascular edema. Severe depletion of lymphoid follicles with proliferation of fibrous tissues (Figure-11) and necrosis in the cellular elements of the splenic corpuscles were also observed. Microscopic lesions of ovine and bovine brucellosis in the uterus revealed partial destruction of the epithelial lining mucosa, granuloma-like structures in the lamina propria, atrophied uterine glands, and other uterine glands exhibiting necrosis and calcification (Figure-12). This event was accompanied by hemorrhage and edema of the myometrium (Figures-13 and 14). Histopathological examination of the placentae of aborted fetuses revealed multifocal necrosis of the allantochorion and placental trophoblasts as well as necrosis of some cotyledonary villi with accumulation of epithelial debris, inflammatory cells, and thrombosis in blood vessels (Figure-15).

### Genotoxicity assessment

**Figure-16:**
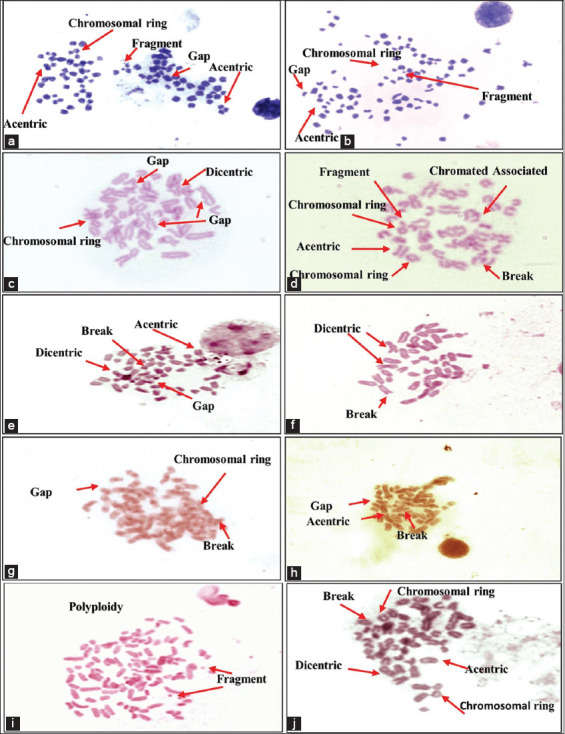
Genotoxicity assessments of *Brucella melitensis* biovar 3 in infected animal species in whole blood leukocytes. Camel (a and b), Buffalo (c and d), Cattle (e and f), Sheep (g and h), and Goat (i and j). All images were taken at 100× magnification.

Eight types of chromosomal aberrations were detected in peripheral blood leukocytes: gaps, breaks, acentric, ring, fusion, dicentric, fragments, and polyploidy (Table-7 and Figure-16). The differences between species were significant in the BCS classification groups. The highest frequency was a dicentric aberration, with 0.025% (25/1000) recorded in low-BCS cattle. In contrast, the lowest frequency was 0.009% (9/1000) recorded for acentric, ring, fusion, and polyploidy in buffaloes high-BCS and cattle [moderate-BCS and high-BCS] (Table-7 and Figure-16). The highest percentage 14.2%, (142/1000) of chromosomal aberrations was recorded in cattle and camels with low-BCS, whereas the lowest percentage was recorded in 8.4% (84/1000) of high-BCS buffaloes (Table-7 and Figure-16).

**Table-7:** Chromosomal aberrations recorded in studied animals’ population with regards to body conditions scores.

Animal species	Body condition score	Total no. of studied cells	Chromosomal aberrations									

			Gaps	Breaks	Acentric	Ring	Centromeric fusion	Dicentric	Fragments	Polyploidy	No.	%
Cattle	Low	1000	21	20	20	15	10	25	21	10	142.00	14.2
	Moderate	1000	20	15	10	12	9	18	15	9	108.00	10.8
	High	1000	14	12	10	11	9	14	15	9	94.00	9.4
Buffalo	Low	1000	20	15	10	10	12	11	12	15	105.00	10.5
	Moderate	1000	11	10	10	10	12	12	14	11	90.00	9
	High	1000	10	10	9	9	10	12	12	12	84.00	8.4
Sheep	Low	1000	15	15	15	15	17	14	20	10	121.00	12.1
	Moderate	1000	11	20	10	10	12	12	14	12	101.00	10.1
	High	1000	11	10	10	13	15	12	12	12	95.00	9.5
Goat	Low	1000	20	15	10	14	18	15	15	20	127.00	12.7
	Moderate	1000	12	12	10	15	20	15	12	13	109.00	10.9
	High	1000	11	10	10	12	16	12	14	11	96.00	9.6
Camel	Low	1000	22	20	22	15	18	14	15	16	142.00	14.2
	Moderate	1000	11	15	18	14	12	12	15	15	112.00	11.2
	High	1000	11	12	11	11	10	12	14	11	92.00	9.2

## Discussion

The prevention and control of brucellosis are interdisciplinary challenges worldwide, which have led to substantial economic losses in animals and humans [1, 2]. Political and health authorities have made persistent efforts to eradicate brucellosis through vaccination and an intensive testing and slaughter strategy [22, 40–42]. Live-attenuated vaccines are effective in inhibiting animal brucellosis. However, this vaccine had its inopportuneness and occasionally retained some virulence in pregnant dams exposed to abortions and *Brucella* excretion through milk; therefore, virulent biohazards for humans interfering in classical serological diagnostic tests [1, 42, 43]. Nonetheless, continued accurate surveillance and efficient infections’ prevention programs among workers at risk of exposure, breeders and their families, veterinarians, laboratory personnel, and dairy and slaughterhouse workers are obligatory [1, 4]. A confirmatory test for the control or eradication of brucellosis must be economical, capable of testing large numbers of serum, and technically appropriate for infected areas. A presumptive diagnosis provided by serological tests is usually accepted as an indication of brucellosis [8, 13]. BAPAT, RBPT, and CFT were used in this study to detect antibodies specific to *Brucella* spp. [44].

Serological screening of sera from 3000 animals of different species using BAPAT (6.1%, 182/3000) in the present study revealed 7.3% (44/600), 4.7% (28/600), 9% (54/600), 6.7% (40/600), and 2.7% (16/600) *Brucella-*positive samples in the investigated cattle, buffalo, sheep, goat, and camel populations, respectively. These results are not significantly different from those of previous studies by Sabour *et al*. [13], Ali *et al*. [26], Abnaroodheleh *et al*. [45], and Hosein *et al*. [46]. BAPAT is still recommended for its high sensitivity despite its moderate specificity, hence it can be applied in the field or in less advanced laboratories [44]. Regarding sensitivity, BAPAT detected more positive reactors 6.1% (182/3000) than RBPT 5.6% (168/3000), indicating that BAPAT is more sensitive than RBPT as an initial screening diagnostic test. The same conclusion was also noted by Dal *et al*. [47], who reported that BAPAT as a presumptive test is recommended because of its higher sensitivity and specificity than that of RBPT; nevertheless, a relatively lower percentage of positive reactors was detected by RBPT 5.6% (168/3000) than by BAPAT 6.1% (182/3000) in this study. This could be explained by the fact that RBPT antigens have higher acidity (pH 3.65), which inhibits the activity of immunoglobulin M (IgM) and enhances the agglutination of IgG only. In contrast, BAPAT antigens have a slightly higher pH (pH 4) that permits the detection of both IgM and IgG (even IgG1) that do not agglutinate at neutral pH but exhibit activity at BAPAT (pH 4) [47]. The RBPT in the present study revealed 6.5% (39/600), 4.3% (26/600), 8.7% (52/600), 6% (36/600), and 2.5% (15/600) seropositive results for the examined cattle, buffalo, sheep, goat, and camel samples, respectively (Table-3). Higher positive percentages of RBPT are justified by its wide detection limits, including IgG1 and IgM antibodies attached to the lipopolysaccharide (LPS) surface antigen of smooth *Brucella* [48]. However, these results do not support RBPT as a confirmative test for the diagnosis of brucellosis. Despite this, RBPT is known to have many false positive and/or negative results due to cross-reactivity between these antigens and other bacterial species, including *Y. enterocolitica* serotype O:9 and *E. coli* serotype O:157 [49], but it is generally simple, rapid, and internationally acknowledged for screening brucellosis in ruminants [2, 12]. Despite its practical drawbacks of time and the need for standardization, CFT is considered a high-quality gold-standard serological test for brucellosis when used correctly. This is advantageous because of its ability to detect IgG1 specifically for *Brucella* infection at low concentrations [50]. Therefore, a good balance between sensitivity and specificity can overcome false results due to cross-reactivity with similar Gram-negative bacteria [2]. In the present study, the prevalence rates of brucellosis using the CFT were 5.8% (35/600), 4% (24/600), 8% (48/600), 5.5% (33/600), and 2.3% (14/600) in cattle, buffaloes, sheep, goats, and camels, respectively, and overall prevalence was 5.1% (154/3000) (Table-4). These results were similar to those of RBPT (Table-3), which coincided with Alamain and Dadar [51], who demonstrated that RBPT and CFT are effective for the detection of *Brucella* spp. antibodies. The CFT and RBPT are recommended by the WOAH for testing animals for international trade. In addition, the WOAH recommended that CFT should be approved worldwide as a confirmatory test for brucellosis identification during control and eradication programs [2]. The high prevalence of brucellosis due to *B. melitensis* biovar 3 in this study among different ruminants may be attributed to (1) keeping sheep and goats nearby or inside households, which is the primary risk factor for household cattle and buffaloes tested serologically positive for brucellosis; (2) dumping abortion-related materials into water canals and housing aborted animals; (3) household owners are unlikely to report suspected cases to the authorities, fearing inappropriate compensation; (4) the presence of an open live animal market near the study area, which facilitates contact of *Brucella*-infected animals with healthy ones; no restriction on animal movement between governorates and infected areas; and (5) lack of biosafety measurements in households and farms [13, 18, 26, 45, 46]. Finally, villagers’ lack of awareness, unfavorable attitudes, and abusive behaviors can increase the risk of brucellosis transmission to animals and humans [2]. Nonetheless, cattle may act as a reservoir for *B. melitensis* biovar 3 in the absence of small ruminants, spreading it to other cattle and resulting in spillover of the pathogen [18]. Eradication of brucellosis through testing and slaughtering seems unfeasible in developing countries because of the limited resources available to compensate farmers whose animals are slaughtered during such screening programs [26, 46].

Bacterial isolation for the diagnosis of brucellosis requires a well-structured laboratory with a biosafety level 3 and highly qualified staff for handling these microorganisms [2]. This study isolated *Brucella* colonies from different internal organs of 60.389% (93/154) seropositive ruminants. Isolation failure in some serologically positive cases 39.61% (61/154) was related to a low viable *Brucella-*load in tissue samples or bacterial contamination; nevertheless, the fastidious nature of *Brucella* spp. [13]. The typing of the 93 *Brucella* isolates involved a combination of growth characteristics, as mentioned previously, and these were the gold-standard identifiers for *B. melitensis* biovar 3 [2]. These results agree with those previously published by Abdel-Hamid *et al*. [18] and Basham *et al*. [52], who isolated *B. melitensis* from different animal species and governorates in Egypt. The study confirmed active brucellosis in the animals tested, which was mainly attributable to keeping ewes, goats, and cattle in the same flock; moreover, it could not be isolated during parturition or abortion [2]. Previously, *B. melitensis* biovar 3 was considered the most prevalent type in Egypt [45, 53]. In addition, Abd-El Halim *et al*. [53] isolated *B. melitensis* biovar 3 from 3 of 87 (3.45%) aborted buffaloes. *B. melitensis* is a pathogenic species found in small ruminants. Therefore, close contact between sheep, goats, cattle, buffaloes, and camels triggers transmission to larger species. Holt *et al*. [54] reported that the main risk factor for inter-species transmission of *B. melitensis* biovar 3 in both large and small ruminants in Egypt is the mixed-rearing system.

The mPCR is a low-risk differential diagnostic technique that requires only the manipulation of genetic material from the pathogenic agent in the laboratory. It is mandatory for brucellosis, the most common occupational disease in laboratories, to be cultured on specific media [55, 56]. The time taken for bacterial characterization by the molecular method is five times shorter than that of the bacterial isolation method [14]. It overcomes the disadvantages of biohazard handling, which reduces the risk of contamination and the cost of determining the final diagnosis [56]. In this study, 93 *Brucella* spp. isolates from cultured tissue homogenates were identified as *B. melitensis* biovar 3 using AMOS-PCR (Figure-3). These results coincided with those of Bricker and Halling [14], who demonstrated the efficiency of a mPCR assay (Bruce-ladder) to differentiate all classical *Brucella* species in seven laboratories using 625 *Brucella* strains from different animal species and geographical origins in a single step.

In this analysis, the biochemical and metabolic panel profiles were biomarkers of both pathophysiology of animal’s body and infectivity of herds. Elevated serum levels of total proteins, albumin/globulin ratio, AST, ALT, urea, urea nitrogen ratio, haptoglobin, and zinc but lower albumin, SOD, and GPx were indicative of cellular leakage, loss of integrity and functionality of the cell membrane in the liver and kidneys, diminished glomerular filtration rate, malnutrition and malabsorption syndrome, and DNA damage, which could be explained by the variation in clinical symptoms and bacterial load between the groups. These were reported to be most significant in the low-BCS group despite their immune reactivity, which was interpreted from their high globulin records [57, 58].

Brucellosis is correlated with oxidative stress biomarkers release, trace elements exhaustion, and acute-phase protein concentrations; therefore, it is indicative of early acute infection. Subsequently, during the chronic stage, a slight increase in serum acute phase proteins concentrations was observed [57, 58]. The present results agree with previous reports stating that the differences in the response and time schedule of concentration are related to the infectious agent type and the kinetics of the measured APPs [57, 58]. Haptoglobin and zinc have been identified as anti-inflammatory, immunomodulatory, and antioxidant modulators, which prevent oxidative tissue damage caused by acute brucellosis in seropositive animals [58, 59]. However, the insignificant changes compared with seronegative ones indicated chronic infectious disease, wasting syndrome, inferior nutrition, and/or low-BCS [58, 60, 61]. In addition, leukocyte binding to fibrinogen through integrin alpha (M) beta 2 (Mac-1) on stimulated monocytes and neutrophils [31, 62] justified the decrease in fibrinogen concentration in *Brucella* seropositive compared with seronegative individuals [58, 60]. In agreement with the current results, SOD activity was lower in *Brucella* seropositive patients during acute infection because of endogenous SOD-A, detoxification of endogenously generated peroxides from metabolism, and inhibition of cytokine activation [58, 62]. In contrast, *Brucella* SOD-C detoxifies exogenous peroxides generated in *Brucella*-seropositive phagocytes [63]. Therefore, glutathione activity increases in response to lipid peroxidation, whereas lower levels are associated with increased catalase activity to overcome bacterial toxins [63]. Similarly, the reported decrease in GPx activity could be explained by fever, decreased appetite, and low-BCS [58]. On the other hand, immunological studies indicated its depletion and/or reduced bioavailability, which was documented during brucellosis oxidative stress to scavenge free radicals in infected seropositive animals [58, 62, 63]. Finally, the estimated acute-phase proteins (Hp and ferritin), oxidative stress biomarkers (SOD, GPx, and NO), and trace elements (iron, copper, and zinc) were useful supplementary indicators that could determine the infection status and clinical stage (acute, sub-acute, or chronic) of brucellosis [58, 62, 63]. Moreover, they were positively correlated with the high frequency of dicentric, polyploidy, and gab-and-break chromosomal aberrations, leading to DNA damage reported among the investigated animal species. Nevertheless, these aberrations increased in frequency due to inferior nutritional conversions (low-BCS) and decreased trace element and oxidative stress levels [35–37]. Hence, oxidative stress biomarkers (SOD, GPx, and NO) and trace elements (iron, copper, and zinc) play an important role in overcoming chromosomal and DNA damage, but they are highly stable during mitotic division [30, 60, 64, 65]. To the best of our knowledge, this is the first report of chromosomal aberrations associated with brucellosis in sheep, goats, buffaloes, cattle, and camels.

In this study, the pathological examination of naturally infected cows was similar to that of naturally infected sheep. These results agree with Meador *et al*. [66], who reported lesions similarities between infected goats and sheep and those in naturally infected cows. These pathological changes were attributed to the continuous exposure of animals to microorganisms and their intermittent dissemination into the blood circulation from old lesions [66]. Similar findings were reported by Shakir [67], who observed lymphoid depletion in the white pulps of the spleens of animals with natural *B. melitensis* infection. Moreover, mild lymphoid depletion in splenic nodules in experimentally infected mice was observed by Celli [68] and Wang *et al*. [69]. The depletion of lymphoid cells and proliferation of reticuloendothelial cells in the spleens and lymph nodes were attributed to their designated immunological functions in the body^’^s vascular compartment by T cells, dependent IgM antibody production, excitation of phagocytosis, and subsequent opsonization against bacterial polysaccharides [68]. Moreover, the reported changes in reticuloendothelial cells during the course of infection, from onset to resolution, provided insights into the ability of *Brucella* cells to stimulate host responses and revealed the localization pattern of organisms in these organs [65, 69]. Elshazly *et al*. [70] reported that the cellular nature of brucellosis lesions consists of inflammatory cells, mainly macrophages, lymphocytes, and plasma cells. Lymphoid depletion occurred after immunodeficiency, as exhibited by *Brucella* growth and log increases within the lymphocytes and macrophages of infected animals, as explained by Tupik *et al*. [71]. Moreover, a few neutrophils were obtained, whereas a large number of lymphocytes associated with histiocytes or macrophages in the form of a diffuse loose granulomatous reaction with a lack of giant cells were also noted as sequel to brucellosis. These results are supported by Yunna *et al*. [72] and Li *et al*. [73], who attributed the presence of a monocyte population to secondary internalization of bacteria. In addition, lymph node granuloma-like structures consist of various types of inflammatory cells, such as lymphocytes and monocytes. The chronic granulomatous lymphadenitis observed in this study coincides with the results obtained by Orecchioni *et al*. [74], who reported collagen fibrotic proliferation and a compact granuloma structure preventing *Brucella* dissemination. Microscopic lesions of ovine and bovine uteri, partial destruction of epithelial lining mucosa, granuloma-like structures in the lamina propria, and calcified necrose-atrophied uterine glands, accompanied by hemorrhage and edema in the myometrium agreed with those previously reported by Meador *et al*. [66] and Huy *et al*. [75]. The occurrence of granulomatous lesions indicates the chronicity of the condition and reflects persistent infection. These results were also explained by Jiao *et al*. [76], who reported the release of proinflammatory cytokines such as tumor necrosis factor-alpha required for the influx of phagocytes to the site of *Brucella* infection for granuloma formation and macrophage activation. Regarding aborted fetal placental multifocal necrosis (allantochorion, trophoblasts, and cotyledonary villi), with the accumulation of epithelial debris and inflammatory cells with thrombosis in blood vessels; these results agree with Rehyan *et al*. [77] observations. Previous reports on *Brucella abortus* indicated that it was carried by the bloodstream to the periphery of the caruncle at the maternal villi capillaries and released into the narrow space adjacent to the fetal erythrophagocytic trophoblast of the chorion. If *Brucella* organisms do not phagocytose, they invade cells, multiply, and spread to the fetus following the ulceration of trophoblasts and invasion of fetal chorionic villi [66]. Meanwhile, degenerative changes and necrosis of udder tissues and infiltration of inflammatory cells are attributed to *Brucella*-infected macrophages undergoing oncosis; a pre-lethal pathway leading to cell death characterized by cell organelle swelling and bulbing, leading to increased cell permeability [78]. In contrast, Shakir [67] showed that *Brucella* is incorporated into phagosomes and remains in the membrane-bound compartment until the host cell dies. The ability of *Brucella* to survive in the intracellular environment is apparently due to the inhibition of phagosome-lysosome infusion, which allows the pathogen to invade phagocytic and non-phagocytic host cells [79]. On the other hand, in a variety of cell types, the potent cytokine stimulatory properties of interleukins (IL-12) and tumor necrosis factor-alpha production by *Brucella* infection may explain the correlation between tissue invasion and localized inflammation [67]. Other lesions indicated the chronicity of *Brucella* infection, which was represented by fibrous invasion and calcification in the udder and uterus [75].

## Conclusion

In conclusion, *B. melitensis* biovar 3 is prevalent in Egypt, with notable seropositive cases identified among ruminant species across multiple governorates. Diagnostic tests revealed varying degrees of seropositivity, highlighting the importance of accurate testing methods. Key clinical signs associated with the disease included mastitis and abortion, although a majority of individuals were asymptomatic.

The control and prevention of brucellosis remain significant interdisciplinary challenges. Accurate diagnosis, particularly in carrier animals, is a critical limitation in current control programs. Molecular technologies, such as AMOS-PCR, have demonstrated high specificity and sensitivity, particularly for detecting carriers. Furthermore, serum biomarkers and oxidative stress indicators showed potential as comprehensive tools for assessing infectious status in relation to body condition and clinical signs, though further optimization is necessary.

Early detection through highly sensitive and specific diagnostic technologies is urgently needed to enhance targeted prophylaxis. The adoption of molecular methods as an alternative to traditional serological techniques should be considered, pending recommendations from international regulatory bodies such as WOAH.

## Data Availability

Supplementary data can be available from the corresponding author upon a reasonable request.
